# Synthesis of mesoporous SnO_2_/NiO nanocomposite using modified sol–gel method and its electrochemical performance as electrode material for supercapacitors

**DOI:** 10.1038/s41598-020-67990-8

**Published:** 2020-07-03

**Authors:** Bhaskar Varshney, M. J. Siddiqui, A. Hakeem Anwer, M. Zain Khan, Faheem Ahmed, Abdullah Aljaafari, Hassan H. Hammud, Ameer Azam

**Affiliations:** 1 Department of Electronics Engineering, Z.H. College of Engineering and Technology, Aligarh Muslim University, Aligarh, Uttar Pradesh 202002 India; 2 Department of Applied Physics, Z.H. College of Engineering and Technology, Aligarh Muslim University, Aligarh, Uttar Pradesh 202002 India; 3 Environmental Research Laboratory, Department of Chemistry, Faculty of Sciences, Aligarh Muslim University, Aligarh, Uttar Pradesh 202002 India; 4 Department of Physics, College of Science, King Faisal University, P.O. Box 400, Al-Ahsa, 31982 Saudi Arabia; 5 Department of Chemistry, College of Science, King Faisal University, P.O. Box 400, Al-Ahsa, 31982 Saudi Arabia

**Keywords:** Porous materials, Electronic properties and materials

## Abstract

In this research work, SnO_2_, NiO and SnO_2_/NiO nanocomposites were synthesized at low temperature by modified sol–gel method using ultrasonication. Prepared samples were investigated for their properties employing various characterization techniques. X-ray diffraction (XRD) patterns confirmed the purity and phase of the samples as no secondary phase was detected. The average crystallite size of the nanocomposites was found to decrease from 19.24 to 4.53 nm with the increase in NiO concentration. It was confirmed from SEM micrographs that the material has mesoporous morphology. This mesoporous morphology resulted in the increase of the surface to mass ratio of the material, which in turn increases the specific capacitance of the material. The UV–Visible spectra showed the variation in the band gap of SnO_2_/NiO at different weight ratio ranging from 3.49 to 3.25 eV on increasing NiO concentration in the samples. These composites with different mass ratio of SnO_2_ and NiO were also characterized by FT-IR spectroscopy that showed shifting of the peaks centered at 545 cm^−1^ in the spectra for NiO/SnO_2_ nanocomposite. The analysis of the electrochemical performance of the material was done with the help of cyclic voltammetry and Galvanostatic charge–discharge. The specific capacitance of the synthesized samples with different concentration of SnO_2_ and NiO was analyzed at different scan rates of 5 to 100 mV/s. Interestingly, 7:1 mass ratio of NiO and SnO_2_ (SN7) nanocomposite exhibited a maximum specific capacitance of ~ 464 F/g at a scan rate of 5 mV/s and good capacitance retention of 87.24% after 1,000 cycles. These excellent electrochemical properties suggest that the SnO_2_/NiO nanocomposite can be used for high energy density supercapacitor electrode material.

## Introduction

As a result of increase in energy consumption demands, conventional energy sources on the earth are getting exhausted day by day. Thus, the whole world has concentrated on energy-related research. The best alternative for the energy is renewable energy. However, the main problem with this type of energy is that it is not available continuously. Hence, it is necessary to store this type of energy when it is available so that it can be utilized whenever needed. Li-ion batteries and supercapacitors are widely used energy storage devices^1–3^. Conventional capacitors have high power density with low energy density and the batteries have high energy density with low power density. A supercapacitor with its high power and cycling is a device that is bridging the gap between the batteries and the conventional capacitors^4,5^. In recent years, lot of research on the electrode material for the supercapacitor has improved the performance of this device^6^. For the electrode material of supercapacitor many transition metal oxides have taken into account and they exhibit high energy performance which is beneficial for supercapacitors development^7–10^. For supercapacitor electrodes, metal oxides behave as promising materials as they show pseudocapacitive effect. Metal oxides such as NiO^11^, SnO_2_^12^, Fe_3_O_4_^13–15^, Fe_2_O_3_^16,17^, Co_3_O_4_^18^, RuO_2_^19–22^, MoO_3_^23^, ZnO^24,25^, CuO^26,27^ and WO_3_^28^ have been developed as promising candidates for supercapacitor electrodes. From these metal oxides, SnO_2_ is a low cost, non-toxic and easily synthesizable nanomaterial^29^. Simple effective sol–gel method for the synthesis of SnO_2_ nanoparticles has been reported by Aziz et al.^30^. SnO_2_ has been used in various applications like photocatalysis, gas sensors and Li-ion batteries^31,32^. SnO_2_ are widely used group among the important oxides that has been used for electrode of supercapacitors^11,33,34^. As NiO can exhibit multiple oxidation states, it favors the condition of fast redox reaction that in turn leads to high specific capacitance^35^ and its composites are widely used in electrochemical applications ^36–40^. Composites made by mixing different metal oxides exhibit very huge electrochemical capacitance^41^. Porous materials are the promising materials for the electrodes of supercapacitor as they provide more active surface for electron transfer and mass transport that in turn results in high specific capacitance.

In the present study nanocomposites of NiO and SnO_2_ were synthesized with different mass ratio by using a modified sol–gel route and their electrochemical properties for the electrode materials for supercapacitors have been investigated. In the modified of sol–gel route, ultrasonication was used for mixing the samples at low temperature leading to a mesoporous structure of the composite material.

## Experimental details

### Materials used

All chemicals were used without further purification. Sodium dodecyl sulphate (SDS) (90%) was purchased from CDH, stannous chloride dihydrate (98%), nickel chloride hexahydrate (97%), ethylene glycol (99%), isopropyl alcohol (99%) and sodium hydroxide (97%) were obtained from Fisher Scientific and methanol (99%) was purchased from S. D. Fine Chemicals.

### Synthesis of SnO_2_ nanoparticles

Firstly, 10 ml distilled water, 5 ml ethylene glycol and 5 ml ethanol were mixed and 1 M SDS was dissolved in the solution. Then 0.1 M stannous chloride dihydrate (SnCl_2_·2H_2_O) was added slowly to the prepared solution and mixed with the help of sonicator in ice bath to maintain low temperature. NaOH solution of 10 M concentration was prepared and then added to the above solution drop wise until the pH becomes 10. Consequently, washing of the sample was done several times by centrifuge. Methanol and distilled water were used for removing ${\mathrm{Cl}}^{-}$ ions and excess NaOH and SDS. The sample was dried using vacuum oven at 100 °C for 24 h and calcined in a furnace at 400 °C for 3 h. Finally, the sample was grinded using agate mortar to get the SnO_2_ nanoparticles.

### Synthesis of NiO nanoparticles

Firstly, 50 ml isopropyl alcohol and 50 ml distilled water were mixed and 0.25 M SDS was dissolved in the solution. Then 0.1 M nickel chloride hexahydrate (NiCl_2_·6H_2_O) was added to the prepared solution and mixed with the help of sonicator in ice bath to maintain low temperature. NaOH solution of 10 M concentration was prepared and then added to the above solution drop wise until the pH becomes 10. Washing of the sample was done several times using a centrifuge. Methanol and distilled water were used for removing ${\mathrm{Cl}}^{-}$ ions and excess NaOH and SDS. The sample was dried using vacuum oven at 100 °C for 24 h and calcined in a furnace at 400 °C for 3 h. Finally, the sample was grinded using agate mortar to get the NiO nanoparticles.

### Synthesis of SnO_2_/NiO nanocomposite

Firstly, 50 ml isopropyl alcohol and 50 ml distilled water were mixed and then 20 g SDS was dissolved in the solution. Then stannous chloride dihydrate (SnCl_2_.2H_2_O) and nickel chloride hexahydrate (NiCl_2_·6H_2_O) in the weight ratio of 1:1, 1:3, 1:5 and 1:7 for SN1, SN3, SN5 and SN7 samples respectively were added to the prepared solution and mixed with the help of sonicator in ice bath to maintain low temperature. NaOH solution of 10 M concentration was prepared and then added to the above solution dropwise until the pH becomes 10. Then washing of the sample was done several times by centrifuge. Methanol and distilled water were used for removing ${\mathrm{Cl}}^{-}$ ions and excess NaOH and SDS. The sample was dried using vacuum oven at 100 °C for 24 h and calcined in a furnace at 400 °C for 3 h. Finally, the sample was grinded using agate mortar to get the SnO_2_/NiO nanocomposite.

### Materials characterization

XRD patterns were collected by Rigaku X-ray diffractometer using radiations of wavelength λ = 1.5406 Å over the angular range of 20° to 80° for SnO_2_/NiO nanocomposite. Intensity of the diffracted radiations was recorded as a function of 2θ. The scanning electron microscopy (SEM) was performed by JEOL JSM-6510LV scanning electron microscope. The morphology of SN1, SN3, SN5 and SN7 nanocomposite was examined by transmission electron microscopy (TEM) (JEOL JSM-2100A). PerkinElmer FTIR spectrophotometer was used to perform the Fourier Transform Infrared Spectroscopy measurements in the wave number ranging from 400 to 4,000 cm^−1^. A PerkinElmer lambda UV–Vis absorption spectrophotometer was used for absorption studies in spectral range of 200–800 nm.

### Electrochemical measurements

For electrode preparation of 80 wt% SnO_2_/NiO nanocomposite, 10 wt% polyvinylidene fluoride (PVDF) and 10 wt% activated carbon were mixed in n-methyl-2-pyrrolidone (NMP) as the mixing media. The mixture was then grinded using mortar to make fine slurry and then it was coated on the tip of a glassy carbon electrode. Electrochemical measurements such as cyclic voltammetry, Galvanostatic charge–discharge (GCD) were performed at room temperature with the help of Metrohm Autolab PGSTAT204N that has three electrode configuration in 2 M KOH electrolyte solution^12^. The glassy electrode was used as working electrode, platinum wire as counter electrode and for reference electrode Ag/AgCl/3.0 M KCl was used.

Galvanostatically charge–discharge was performed at current density of 2.5 and 5 A/g in a potential range of − 1 to + 1 V. CV was carried out at various scan rates in the range from 5 to 100 mV/s. Electrochemical Impedance Spectra (EIS) analyses were performed using frequency range between 0 Hz to 50 kHz and a perturbation voltage of 10 mV/s was used.

## Results and discussion

**Figure 1:**
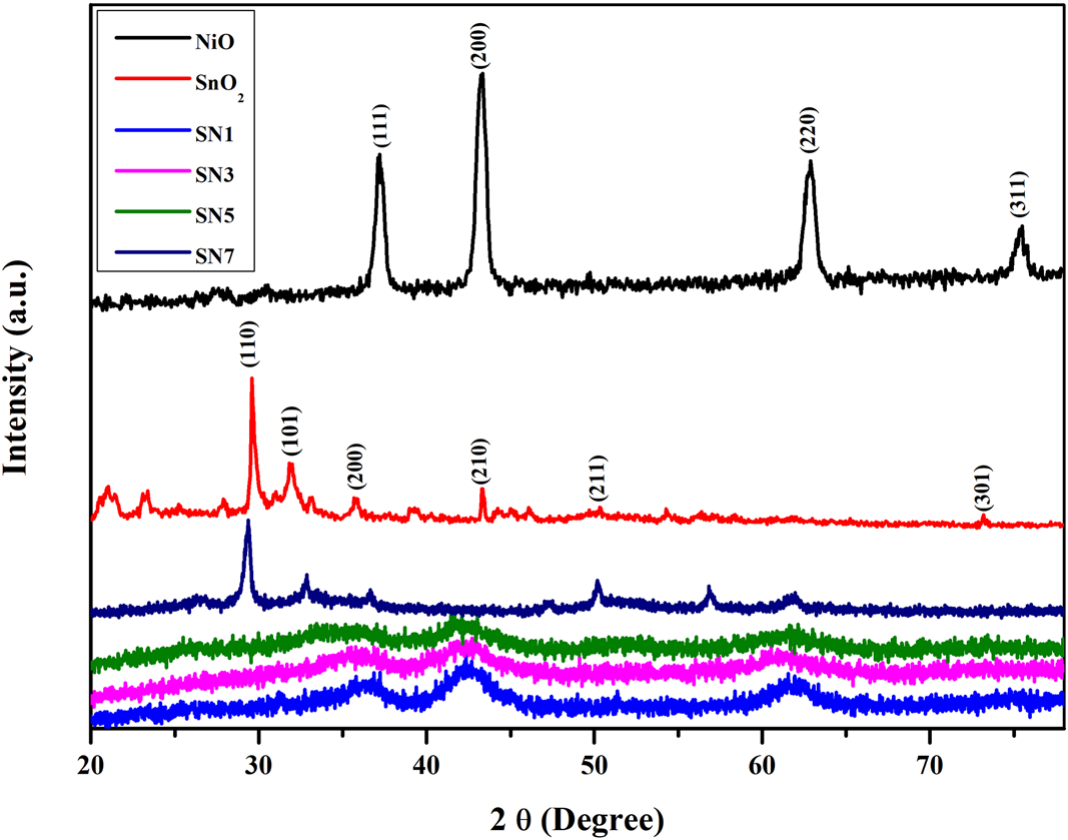
XRD patterns of SnO_2_, NiO, SN1, SN3, SN5 and SN7.

The crystal structures of SnO_2_, NiO, SN1, SN3, SN5 and SN7 nanocomposite were characterized by XRD as shown in Fig. 1. The diffraction peaks of SnO_2_/NiO nanocomposites were found to be at diffraction angle 2θ = 29.4°, 32.89°, 36.7°, 42.35°, 50.2°, 56.85° and 61.68° which are assigned to (110), (101), (111), (200), (211), (002) and (310) crystal planes of the samples, respectively. Using Scherrer’s formula, the average particle size was calculated. The approximate crystallite sizes in the powder samples were estimated to be 48.2, 22.63, 19.24, 13.17, 4.89 and 4.53 nm for SnO_2_, NiO, SN1, SN3, SN5 and SN7, respectively. Two-phase crystal structure has been clearly identified in the XRD diffraction pattern. In the XRD pattern, broad peaks having low intensity describe the tetragonal phase of SnO_2_ and sharp peaks with high intensity describe the cubic phase of NiO^42,43^. On increasing the ratio of NiO in the composite, there is a broadening of peak at an angle ~ 42° that corresponds to NiO, this shows that the attachment of NiO nanoparticles increases on increasing the concentration of NiO in the composite.

**Figure 2:**
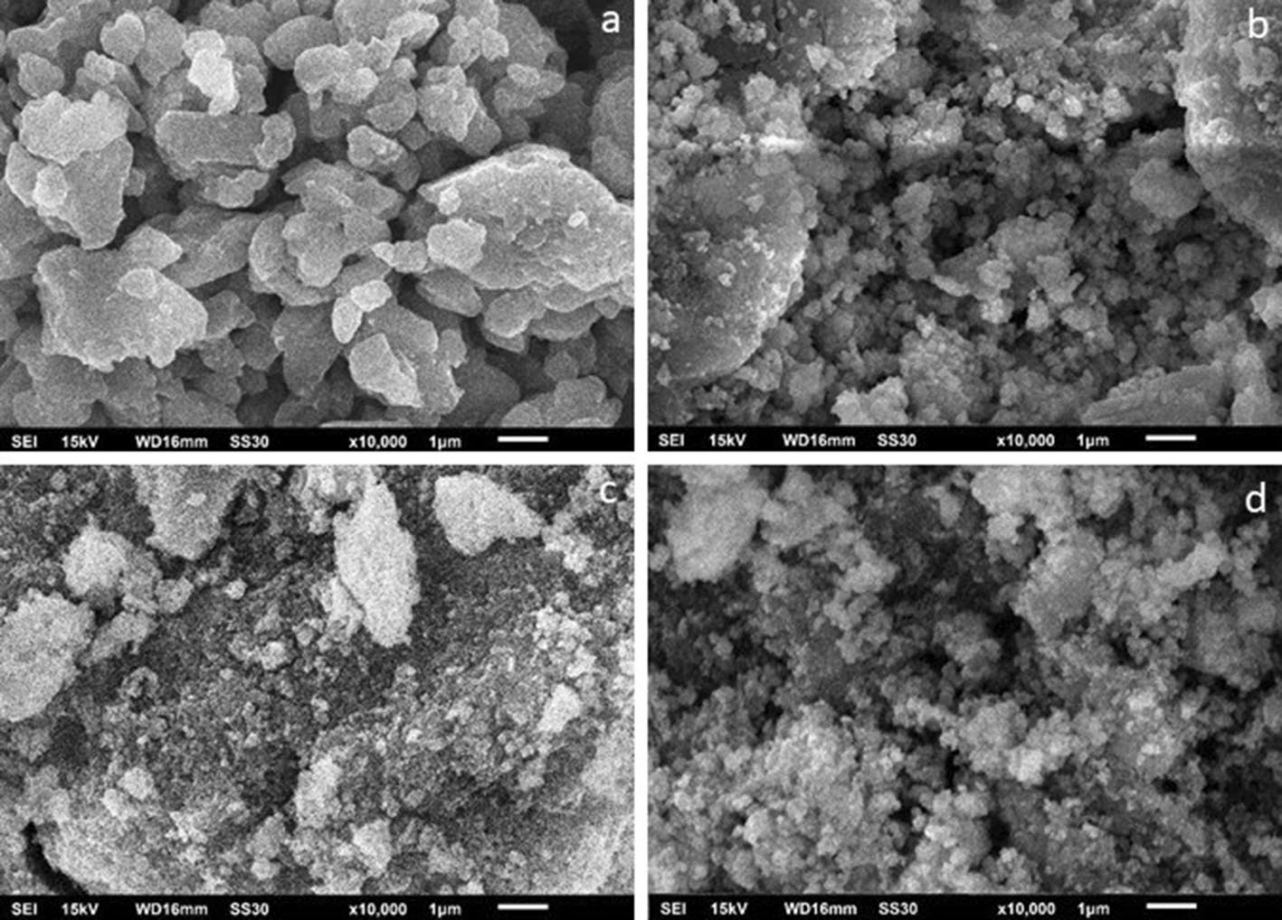
SEM images of (**a**) SN1, (**b**) SN3, (**c**) SN5 and (**d**) SN7.

**Figure 3:**
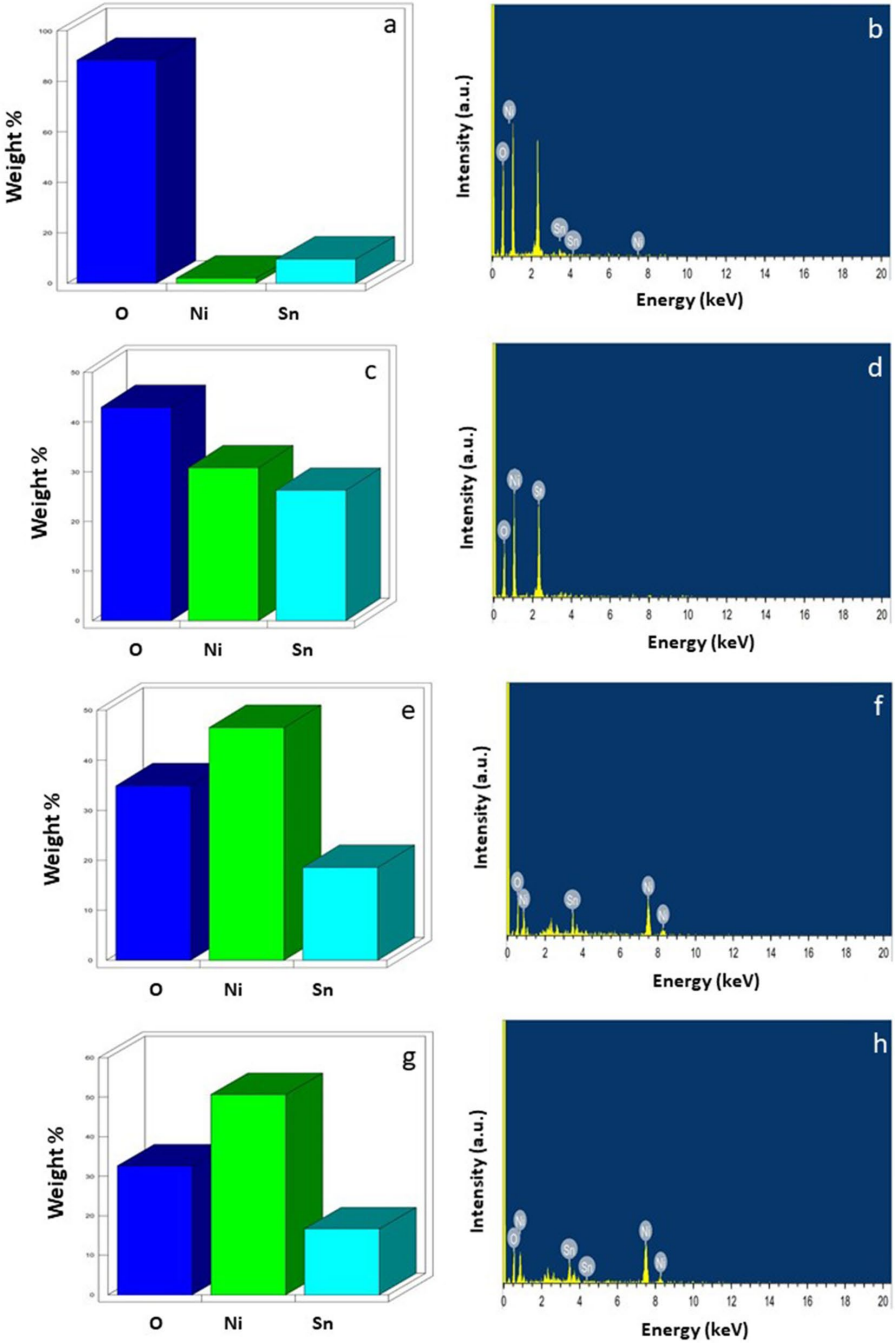
EDS spectra showing the composition of constituent elements Oxygen, Nickel and Tin in (**a**) and (**b**) SN1, (**c**) and (**d**) SN3, (**e**) and (**f**) SN5 and (**g**) and (**h**) SN7.

Figure 2a–d shows the morphology of synthesized nanocomposites SN1, SN3, SN5 and SN7, respectively. The SEM image in Fig. 2 clearly shows that large number of particles are having homogeneous size. The energy dispersive spectroscopy (EDS) results are shown in Fig. 3. Elements oxygen, tin and nickel were present in the mesoporous SN1, SN3, SN5 and SN7, which confirms the presence and formation of SnO_2_ and NiO structures. The EDS analysis exhibited clear peaks of Sn, Ni and O elements, and no additional peaks were observed, which means that the prepared Nano powder is devoid of impurities that arise from the starting precursors like chlorine and carbon.

**Figure 4:**
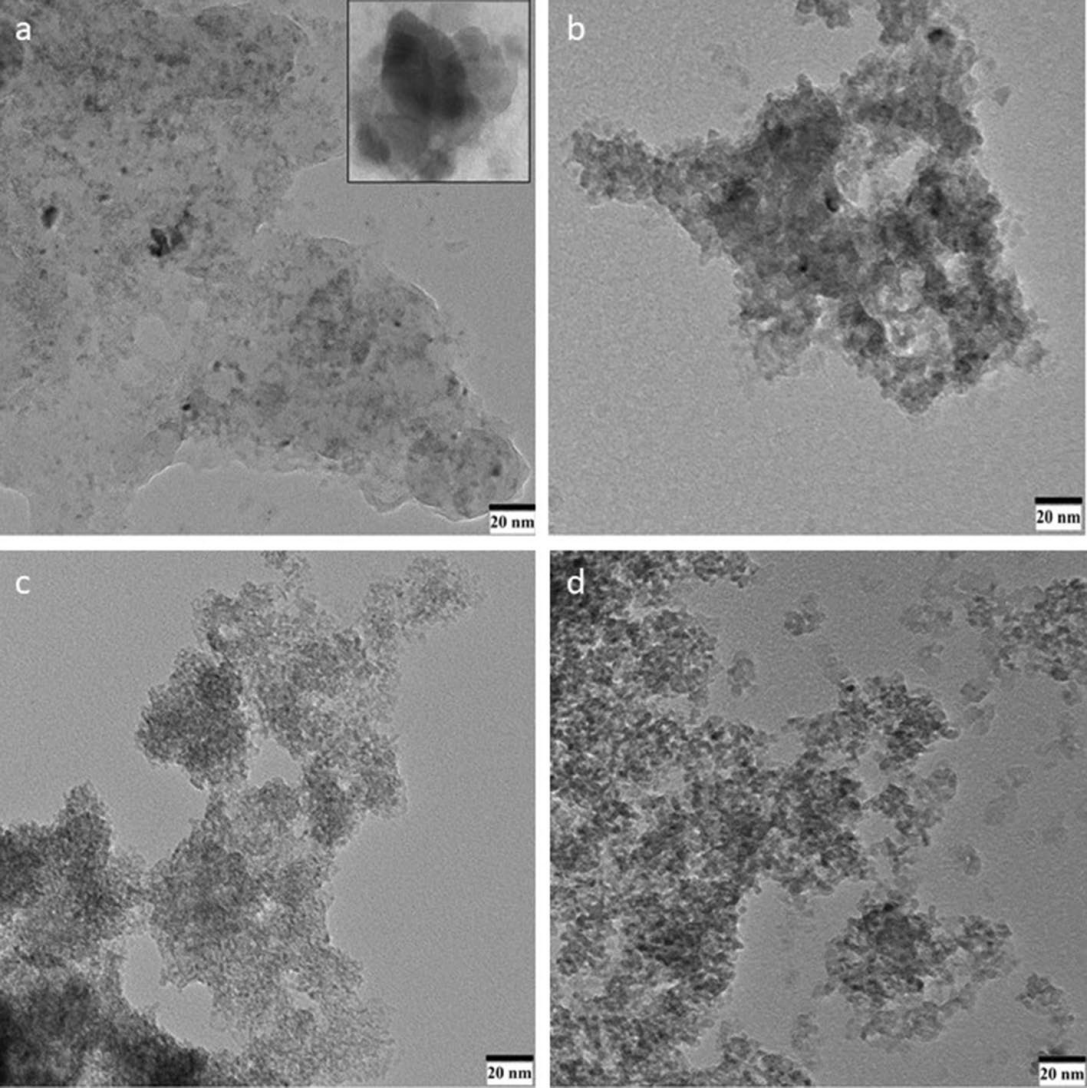
TEM images of (**a**) SN1_,_ (**b**) SN3, (**c**) SN5 and (**d**) SN7.

**Figure 5:**
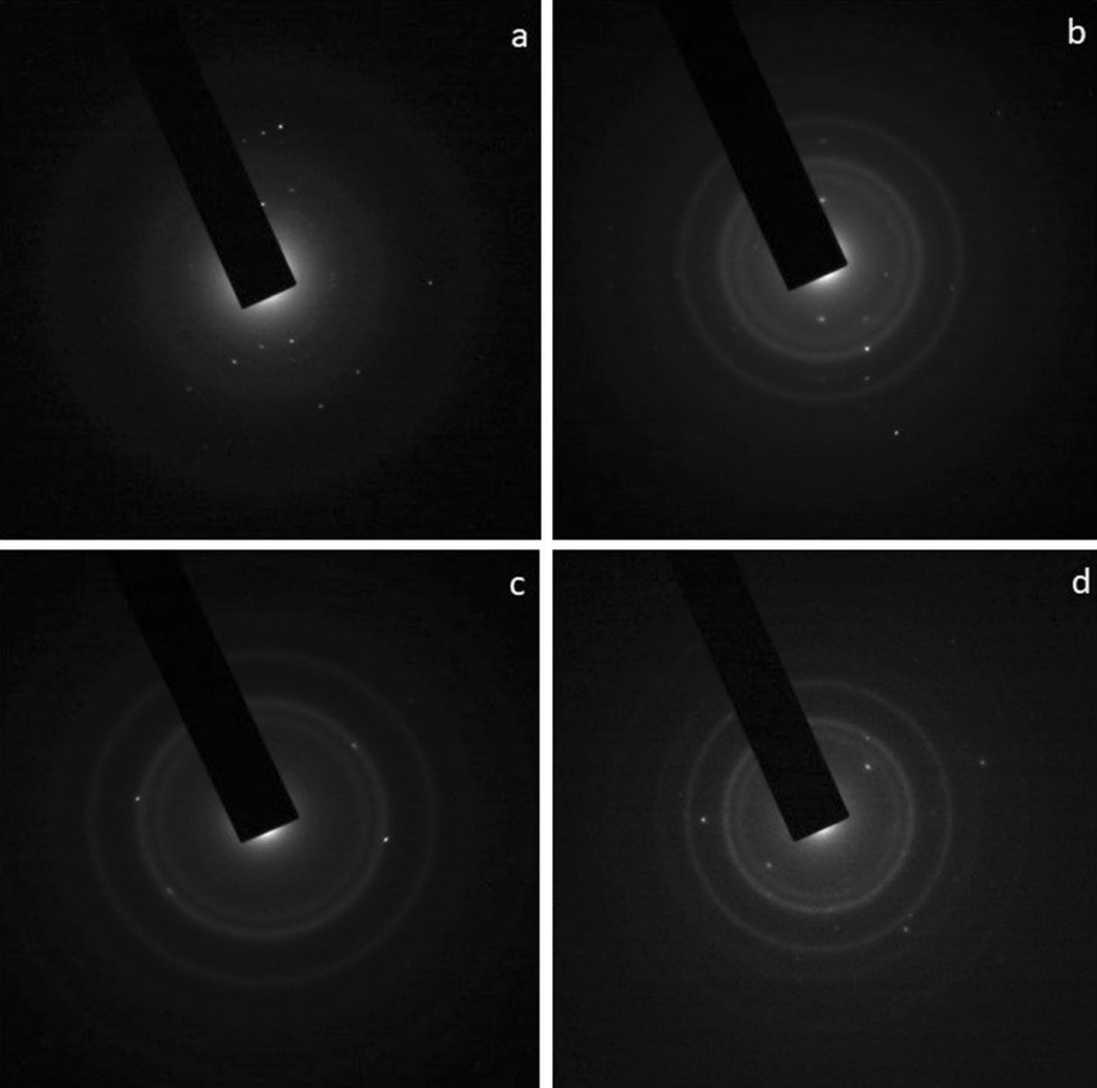
SAED patterns of (**a**) SN1_,_ (**b**) SN3, (**c**) SN5 and (**d**) SN7.

The morphology of SN1, SN3, SN5 and SN7 nanocomposite was examined by the TEM. TEM images have been recorded using a high-resolution transmission electron microscope operating at 200 keV. Figure 4 shows the TEM micrographs of SnO_2_ and NiO nanocomposite, in which clear spherical particles like structure is visible. Figure 4a shows the TEM image of SnO_2_/NiO nanocomposites (SN1) revealing a spherical like structure (~ 20–22 nm) and NiO nanoparticles on the surface of SnO_2_ nanoparticles matrix can be clearly seen. The particle size observed by TEM is in good agreement with that estimated by X-ray line broadening (~ 19 nm for SN1 sample). The NiO nanoparticles were well distributed onto SnO_2_ nanoparticles and the inset shows the distribution at higher magnification. The TEM image also shows the tight contact between NiO and SnO_2_ nanoparticles suggesting the strong interaction between the two substances. It is clear from Fig. 4a–d that correspond to the TEM images of SN1, SN3, SN5 and SN7 respectively, the size of nanocomposites decreased (from ~ 22 nm to ~ 5) with the increase in NiO concentration. The size reduction with the NiO concentration is in good agreement with the XRD studies. Figure 5 shows the selected area electron diffraction (SAED) pattern of SN1, SN3, SN5 and SN7, which confirms the polycrystalline nature of the nanocomposite. Multiple rings are seen in SAED spectrum, as expected from the XRD pattern.

**Figure 6:**
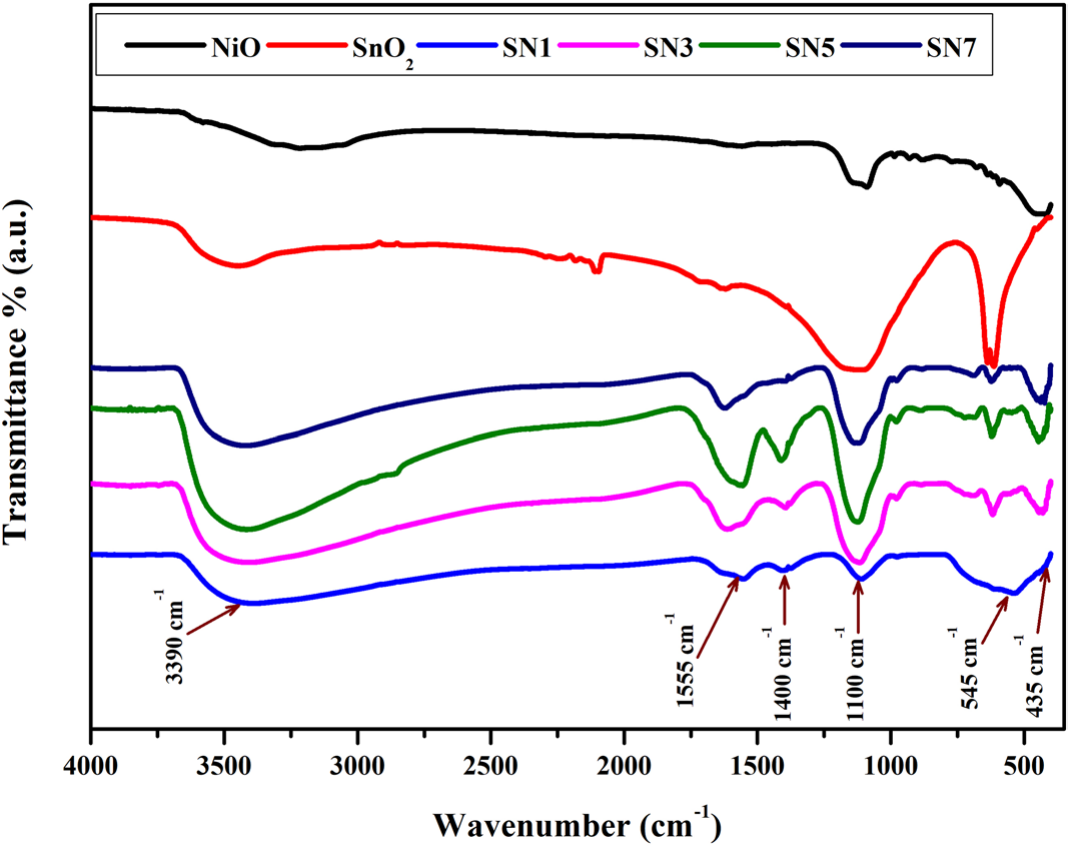
FT-IR spectra of SnO_2_, NiO, SN1, SN3, SN5 and SN7.

Figure 6 shows the FTIR spectra of SnO_2_, NiO, SN1, SN3, SN5 andSN7 nanocomposites. The peak at around 614–930 cm^−1^, which refers to Sn–O stretching modes of Sn–O–Sn, appeared even after calcination at 400 °C. The band around 1,100 cm^−1^ is associated with the C=O. The band around 1,400 cm^−1^ is associated with the stretching bands of COO– group. The band around 3,390 cm^−1^ is associated with the O–H stretching. Shifting of peak was clearly shown in the graph for NiO/SnO_2_ nanocomposite with the peak at 545 cm^−1^^44,45^.

**Figure 7:**
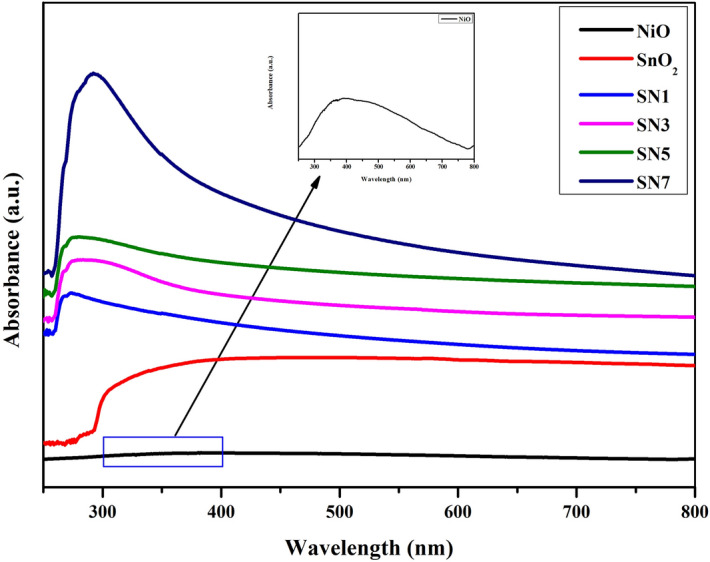
UV–Vis absorption spectra of SnO_2_, NiO, SN1, SN3, SN5 and SN7 and NiO absorbance in inset.

UV–Vis absorption spectra of SnO_2_, NiO, SN1, SN3, SN5 and SN7are shown in Fig. 5. SnO_2_ and NiO nanoparticles shows characteristic peaks at 390 nm and 380 nm respectively as shown in Fig. 7. The absorption peak at 295 nm in the SnO_2_/NiO confirmed the covalent attachment of SnO_2_ and NiO nanoparticles.

**Figure 8:**
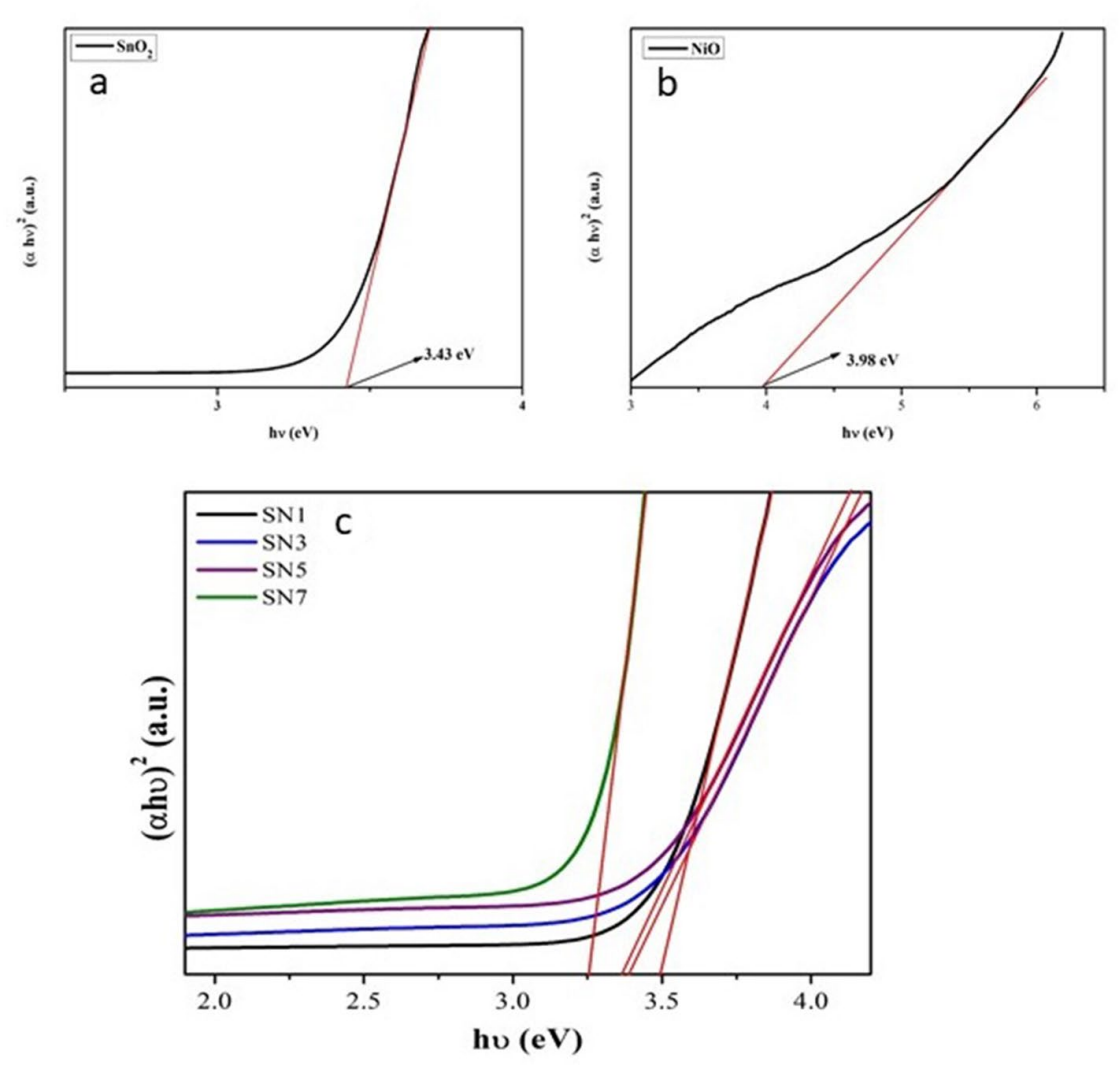
Energy band gap plot of (**a**) SnO_2_, (**b**) NiO, (**c**) SN1, SN3, SN5 and SN7.

The band gap of the samples was calculated using the following equation derived by Tauc’s relation^46^.

1 $$\upalpha {\text{h}}\upupsilon = {\text{K}}\left( {{\text{h}}\upupsilon - {\text{E}}_{{\text{g}}} } \right)^{{\text{n}}}$$

where α is the coefficient of absorption, hυ is the incident light energy, E_g_ is the optical energy band gap and *n* is a number used to characterize the optical absorption processes. *n* = 1/2 for direct transition and *n* = 2 for indirect transition for high absorbing region, where α obeyed the above equation. By plotting (αhυ)^2^ as a function of photon energy hυ (eV) and extrapolating the linear regions of this curve to (αhυ)^2^ = 0 the band gap of the material was determined as shown in Fig. 8.

The value of band gaps in SnO_2_, NiO, SN1, SN3, SN5 and SN7 were found to be 3.43 eV, 3.98 eV, 3.495 eV, 3.39 eV, 3.36 eV & 3.252 eV respectively. On increasing the weight percent of NiO in the composite the band gap of the nanocomposite was found to decrease, even though the particle size of the material decreases. This phenomenon shows the opposite behavior, usually the size of the particle has reverse relation with the bandgap, and size in this study was reduced, so bandgap should increase. In this case, the reason for opposite behavior is due to the increase in weight percent of NiO. This behavior is in good agreement with the results already reported by Chun-Ming et al.^46^ and Azam et al.^47^. Rakhshani et al. proposed that this has been due to the direct indirect transition^48^. Many group has also suggested that the band gap narrowing effect may be due to the impurity phases that arises due to the alloying effect of parent compound^49,50^.

**Figure 9:**
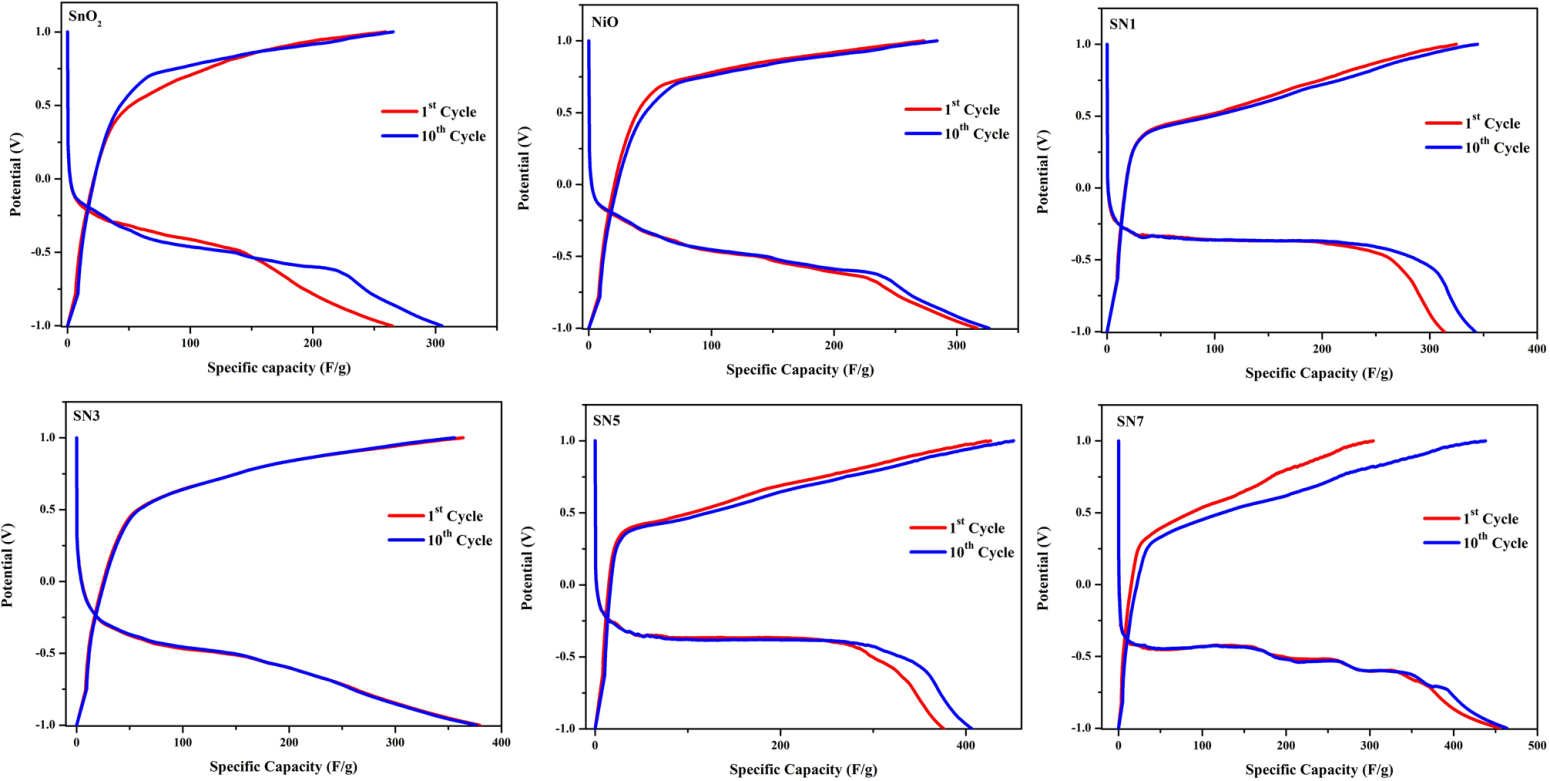
Galvanostatic charge/discharge curve of SnO_2_, NiO, SN1, SN3, SN5 and SN7 for 1st and 10th cycle at a current density of 2.5 A/g.

Figure 9 shows the Galvanostatic charge/discharge curve of SnO_2_, NiO, SN1, SN3, SN5 and SN7 for 1st and 10th cycle from a voltage range of − 1 V to + 1 V at a current density of 2.5 A/g. The specific capacitance is calculated over a limited potential window, hence it changes for wider or narrower potential window. Therefore, in our case the specific capacity has been calculated by multiplying the specific capacitance with width of potential window^51^ as it gives consistent value and can be used for comparison with other materials.

For the calculation of specific capacitance, following formula was used^52^

2 $$C_{s} = \frac{Q}{m\Delta V}$$

Here C_s_ is the specific capacitance of the material (F/g), ∆V is the potential window used (V), *m* is the active material mass (g), Q is the charge stored in the full cycle. The obtained values of specific capacitance (F/g) for SnO_2_, NiO, SN1, SN3, SN5 and SN7 at scan rates of 5, 10, 25, 50 and 100 mV/s are given in Table S1.

**Figure 10:**
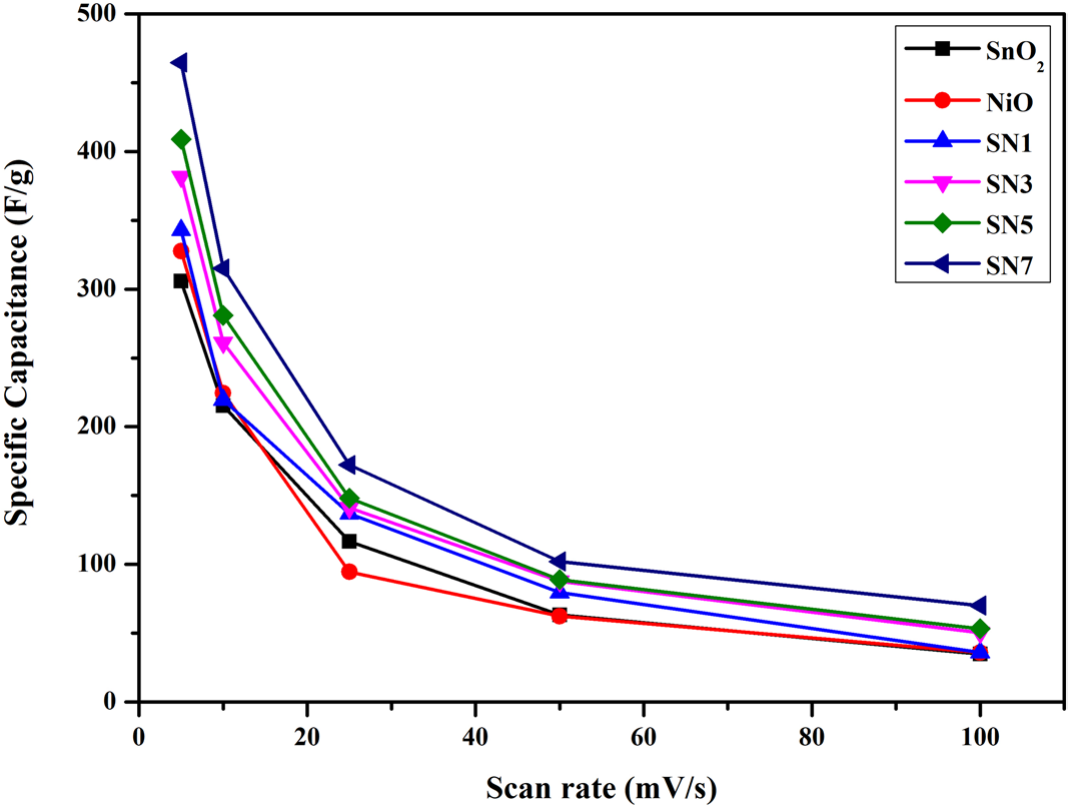
Specific capacitance of SnO_2_, NiO, SN1, SN3, SN5 and SN7 at different scan rates.

Due to the ion exchange mechanism, the specific capacitance of the material decreases on higher scan rates^52^. It is clearly shown from the above data that the weight ratio of NiCl_2_·6H_2_O over SnCl_2_·2H_2_O have remarkable effects on the electrochemical properties of SnO_2_–NiO nanocomposite. It is evident from the data that the value of C_s_ increases on increasing the ratio of Ni over Sn. A maximum value of C_s_ 464.67 F/g was observed for SN7 at scan rate of 5 mV/s. Figure 10 shows the specific capacitance values of SnO_2_, NiO, SN1, SN3, SN5 and SN7 at the scan rates of 5, 10, 25, 50, and 100 mV/s for a potential window of − 1 to + 1 V. From the graph, it can be clearly seen that the value of specific capacitance is decreasing on increasing the scan rate. The maximum value of specific capacitance was obtained at a lower scan rate of 5 mV/s for SN7.

Figure S1 shows the cyclic voltammetry (CV) curves of for SnO_2_, NiO, SN1, SN3, SN5 and SN7 nanocomposites at the different scan rates of 5, 10, 25, 50, and 100 mV/s in the potential window of − 1 to + 1 V. The bend shows a semi-rectangular shape, demonstrating a perfect capacitive behavior. Moreover, the linear increment of the current with an increment of scan rate demonstrates that the charge is essentially non-faradic in nature.

**Figure 11:**
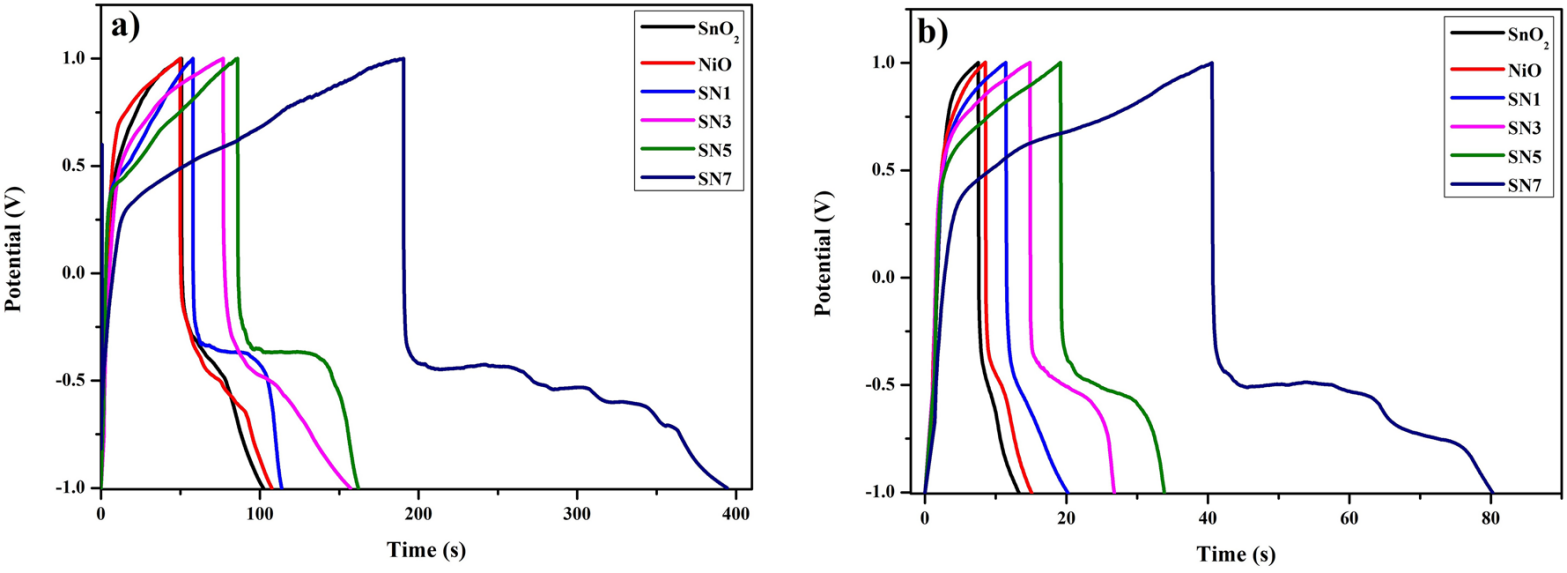
Galvanostatic charge–discharge curves of different samples at (**a**) 2.5 A/g and (**b**) 5 A/g (V–t curve).

The charge–discharge conduct of the SnO_2_, NiO, SN1, SN3, SN5 and SN7 nanocomposites electrode was inspected by Galvanostatic charge release strategy at a consistent current density of 2.5 A/g and 5 A/g in the potential range from − 1 to + 1 V. The obtained values of specific capacitance (F/g) for SnO_2_, NiO, SN1, SN3, SN5 and SN7 at current rates of 2.5 A/g and 5 A/g are given in Table S2. Figure S2(a–f) demonstrates a typical Galvanostatic charge–discharge curve of SnO_2_, NiO, SN1, SN3, SN5 and SN7 nanocomposites electrode. Figure 11a, b shows the Galvanostatic charge–discharge curves of different samples at 2.5 A/g and 5 A/g (V–t curve) respectively. It is clear that the whole curve is linear and symmetrical for all samples, which demonstrates that the electrode has perfect capacitive quality and amazing electrochemical reversibility.

For the calculation of specific capacitance in case of charge–discharge curve the following formula was used

3 $$C_{s} = \frac{i\Delta t}{{m\Delta V}}$$

where *i* is the discharging current, ∆t is the discharging time and m is the loading mass of the sample on current collector. Table S3 shows the different loading mass of the sample taken on the current collector.

From the above data, it has been observed that the specific capacitance of SN7 has the maximum value of 253.94 F/g at the current density of 2.5 A/g (see Table S2) that supports the result observed from CV that is specific capacitance of the SnO_2_–NiO increases on increasing the mass ratio of NiCl_2_·6H_2_O over SnCl_2_·2H_2_O.

**Figure 12:**
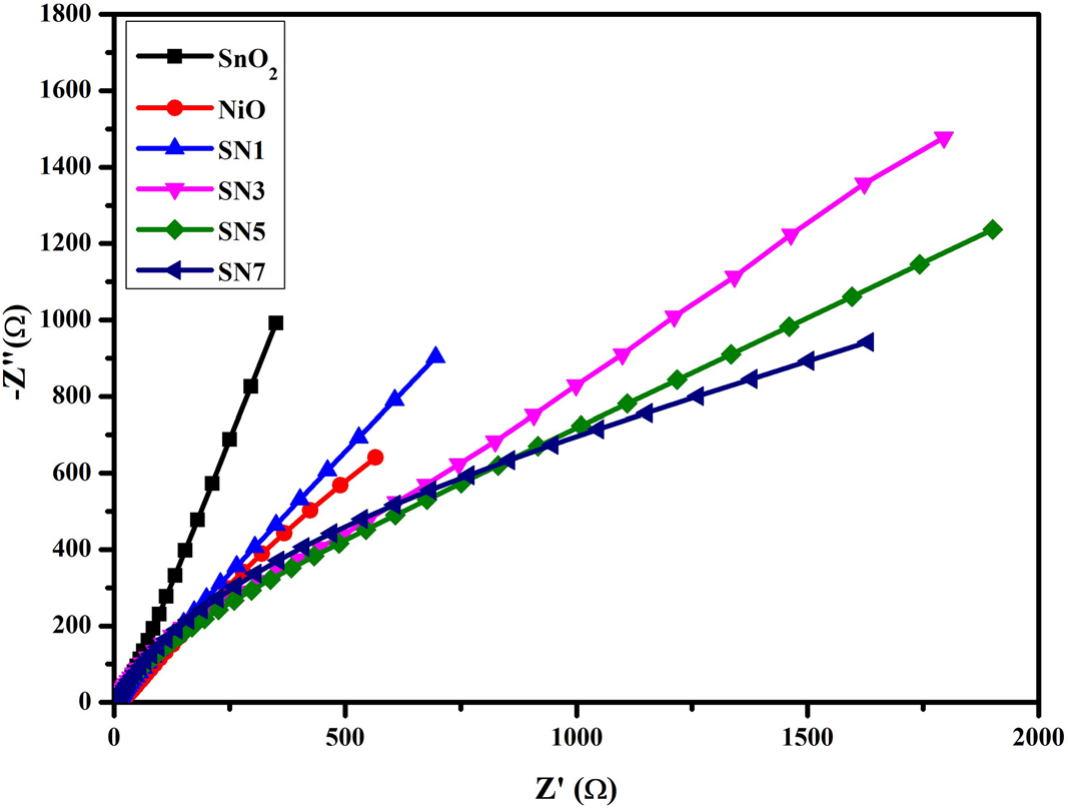
Electrochemical impedance spectra for SnO_2_, NiO, SN1, SN3, SN5 and SN7 from 0 Hz to 50 kHz in the form of Nyquist plots.

Figure 12 represents the electrochemical impedance spectra (EIS) for SnO_2_, NiO, SN1, SN3, SN5 and SN7 nanocomposite in the form of Nyquist plots that were recorded using complex impedance spectroscopy technique. EIS analysis was performed by applying a perturbation voltage of 10 mV/s in a frequency range between 0 Hz and 50 kHz. The impedance spectroscopy helps us to distinguish between the grains and grain boundaries contribution as both have different relaxation time. In general, the grain boundaries (poorly conducting) are more active at lower frequencies whereas the conducting grains are dominant if the frequency of applied field increases^53^. The Nyquist plot shows the presence of a single circular arc for all samples, which reveals the conduction of grain boundaries only. In addition, it can also be observed that impedance increases with increasing weight percent of NiO resulting in decrement of conductivity that corresponds to the increase in specific capacitance.

**Figure 13:**
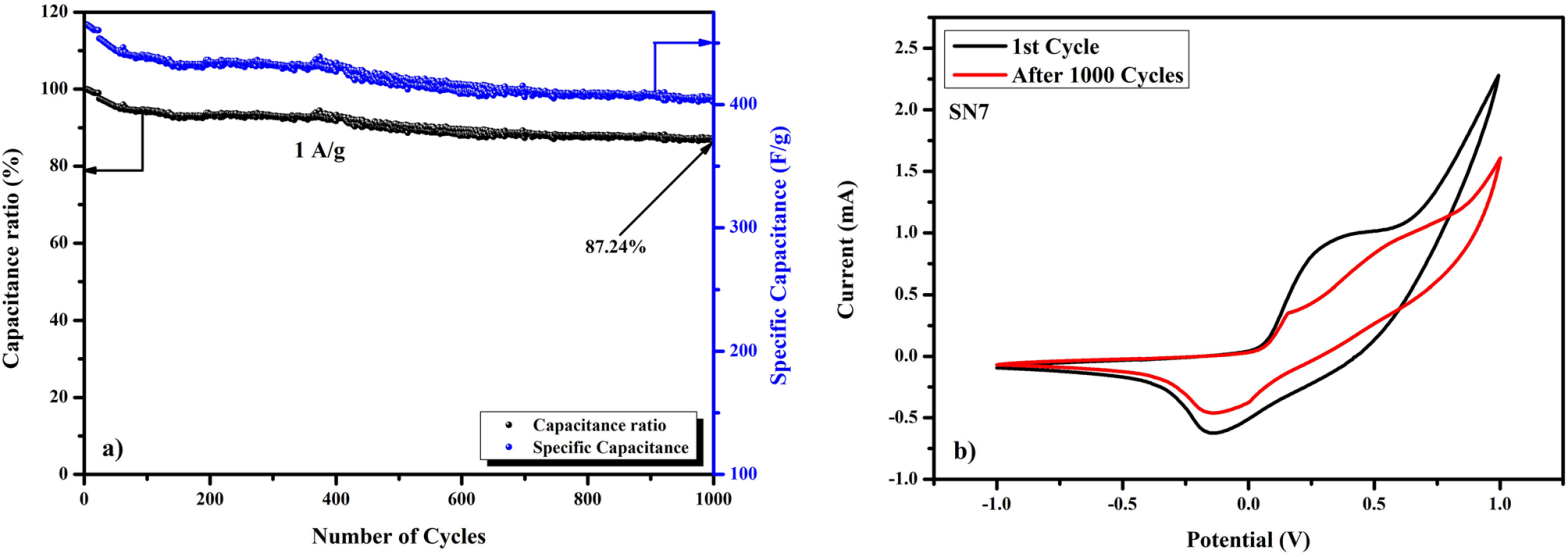
(**a**) Long term cycling stability and (**b**) CV curves before and after cycling.

Long term cycling test has been done to evaluate the cycling stability of the as synthesized sample SN7. Figure 13a shows the cyclic performance at a current density of 1 A/g for SN7 over 1,000 cycles. The value of specific capacitance of ~ 400.16 F/g and a capacitance retention of 87.24% was found to be retained even after 1,000 cycles, which shows a good charge–discharge behavior^54,55^. Zhang et al.^54^ reported that three-dimensional self-assemble polypyrrole@MnCo_2_O_4_ nanoarchitectures on graphite foam shows capacitance retention of 85.3% after 1,000 cycles, in addition there is no loss in capacitance even after 10,000 cycles with capacitance retention of 85.5%. In another report, Zhu et al.^55^ showed that a three-dimensional (3D) free-standing polypyrrole@NiCo_2_S_4_/GF electrode could deliver a high rate performance of 84.4% retention after 1,000 cycles. It is obvious that the material used in our research is different from that of the above mentioned references; instead, the cyclic stability of our supercapacitor electrode material (SnO_2_/NiO nanocomposite) is comparable to the reported results, which clearly indicates that the material used in our research has great potential application for supercapacitor. Figure 13b shows the CV curves before and after cycling test at a scan rate of 20 mV/s, both CV curves are similar with two-redox peaks. However, after cycling, the potential difference is smaller between the oxidation/reduction peaks.

## Conclusion

In summary, a modified sol gel method has been developed for the synthesis of mesoporous SnO_2_–NiO nanocomposites and the nanocomposites so prepared were employed as a supercapacitor electrode. XRD results confirmed that the phase purity and nanocrystalline formation of SnO_2_–NiO nanocomposites and size of the particles depends on the concentration of NiO. These parameters were found to decrease on increasing the concentration of NiO in the samples, which resulted in an increase of specific capacitance. UV–Vis spectra showed a decrease in band gap of the composites on increasing the concentration of NiO that is lowest in case of SN7. SEM images showed that these nanocomposites appear to be spherical in shape and have mesoporous structure. The mesoporous structure of the synthesized material provides a high active surface for electron transfer and results in high specific capacitance. The electrochemical tests showed that the prepared SnO_2_–NiO nanocomposite electrode exhibited an ideal capacitive behavior with a maximum specific capacitance of 464.67 F/g at a scan rate of 5 mV/s. Table 1 shows the comparison of specific capacitance with previously synthesized nanomaterials. A good electrochemical response was observed in terms of specific capacity in Galvanostatic charge–discharge, rate capability, cyclic stability and Coulombic efficiency. This excellent performance is due to the mesoporous structure of the prepared composite. These results demonstrate that the prepared SnO_2_–NiO nanocomposites are suitable candidates and more attractive electrode material for the commercial supercapacitors applications.

**Table 1:** Comparison with previous work.

Sample	Specific capacitance	Reference
SnO_2_	122 F/g at 5 mV/s	^12^
CS@NiO	270.4 F/g at 0.5 A/g	^40^
Hollow SnO_2_ microspheres	178.86 F/g at 1 mV/s	^31^
Mesoporous NiO nanowires	348 F/g at 10 mV/s	^56^
Mesoporous SnO_2_@NiO	464.67 F/g at 5 mV/s	Present work

## Supplementary information

Supplementary file1 (PDF 270 kb)
