# Supplementary material for: Synthesis of mesoporous SnO2/NiO nanocomposite using modified sol–gel method and its electrochemical performance as electrode material for supercapacitors
Source: Sci Rep. 2020 Jul 3;10:11032. doi: 10.1038/s41598-020-67990-8 (PMC7335181; doi:10.1038/s41598-020-67990-8)
Supplement: Supplementary file 1 — Supplementary file1 (PDF 270 kb) [file 41598_2020_67990_MOESM1_ESM.pdf]

Synthesis of mesoporous SnO_2/NiO nanocomposite using modified sol-gel method and its electrochemical performance as electrode material for supercapacitors

Bhaskar Varshney[1, 2], M. J. Siddiqui[1], A. Hakeem Anwer[3], M. Zain Khan[3], Faheem Ahmed[4], Abdullah Aljaafari[4], Hassan H. Hammud[5], Ameer Azam*[2]

1-Department of Electronics Engineering, Z.H. College of Engineering & Tech., Aligarh Muslim University, Aligarh-202002, Uttar Pradesh, India

2- Department of Applied Physics, Z.H. College of Engineering & Tech., Aligarh Muslim University, Aligarh-202002, Uttar Pradesh, India

3- Environmental Research Laboratory, Department of Chemistry, Faculty of Sciences, Aligarh Muslim University, Aligarh-202002, Uttar Pradesh, India

4- Department of Physics, College of Science, King Faisal University, Al-Ahsa 31982, Saudi Arabia

5- Department of Chemistry, College of Science, King Faisal University, Al-Ahsa 31982, Saudi Arabia

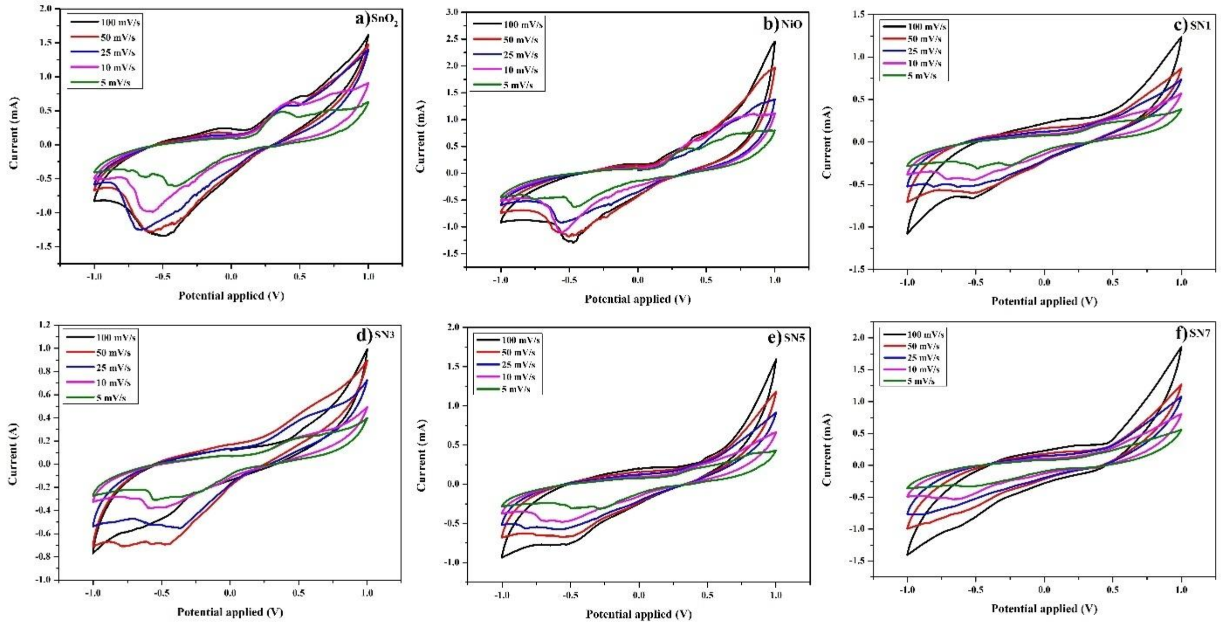

Figure S1: Cyclic Voltammetry curve for a) SnO_2, b) NiO, c) SN1, d) SN3, e) SN5 and f) SN7 at different scan rates

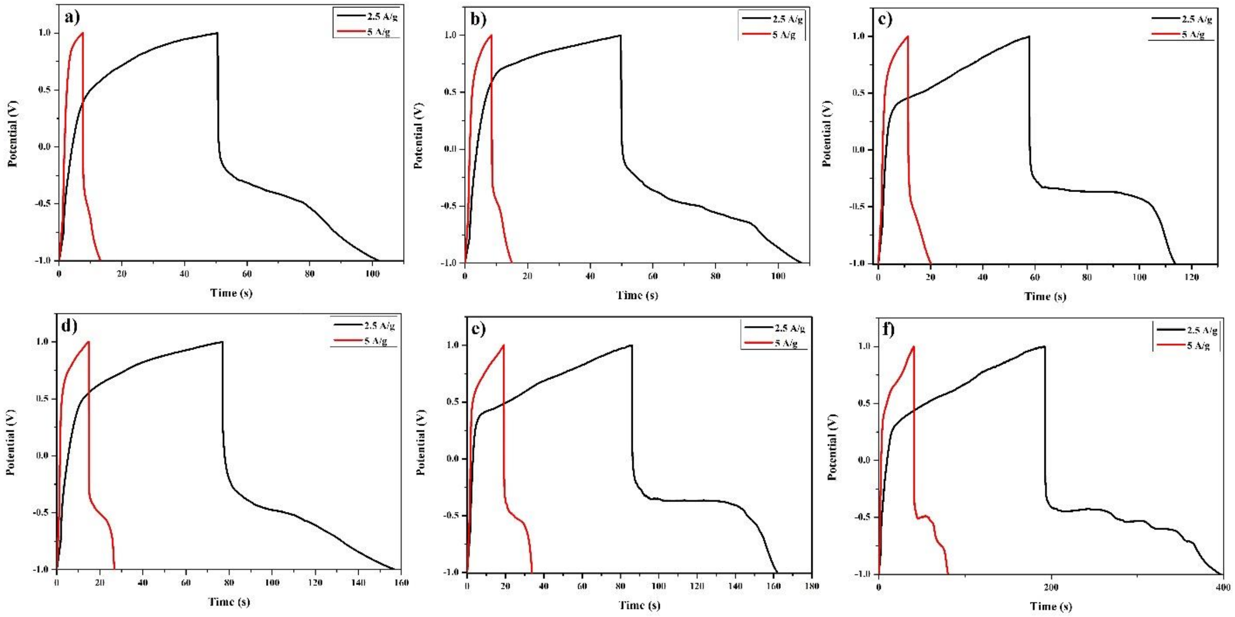

Figure S2: Galvanostatic Charge-Discharge curves for a) SnO_2, b) NiO, c) SN1, d) SN3, e) SN5 and f) SN7 at different current rates

Table S1: Specific capacitance of SnO_2, NiO, SN1, SN3, SN5 and SN7 at different scan rates

**Sample**	**Specific capacitance at different scan rates (F/g)**				
	**100 (mV/s)**	**50 (mV/s)**	**25 (mV/s)**	**10 (mV/s)**	**5 (mV/s)**
**SnO_2**	34.88	63.16	116.62	215.34	305.82
**NiO**	35.96	62.41	94.62	224.63	327.80
**SN1**	36.04	79.72	136.96	219.73	342.94
**SN3**	50.31	87.81	141.04	261.35	381.86
**SN5**	53.18	88.90	148.14	281.04	408.96
**SN7**	70.05	102.11	172.26	315.07	464.67

Table S2: Specific capacitance of SnO_2, NiO, SN1, SN3, SN5 and SN7 at different current densities

**Sample**	**Specific capacitance at different current rates (F/g)**	
	**5 A/g**	**2.5 A/g**
**SnO_2**	15	65
**NiO**	16.75	71.25
**SN1**	22.2	72.83
**SN3**	29.95	99.83
**SN5**	36.85	101.21
**SN7**	98.45	253.94

Table S3: Loading mass of samples on current collector

**Sample**	**Loading mass of sample on current collector (mg)**
**SnO_2**	0.338
**NiO**	0.359
**SN1**	0.160
**SN3**	0.160
**SN5**	0.159
**SN7**	0.159
